# Translating Emergency Knowledge for Kids (TREKK): using research evidence to improve outcomes for children and families in emergency departments across Canada

**DOI:** 10.1007/s43678-021-00205-9

**Published:** 2021-10-08

**Authors:** Lisa Knisley, Lisa Hartling, Mona Jabbour, David W. Johnson, Eddy S. Lang, Shannon MacPhee, Sarah Reid, Shannon D. Scott, Doug Sinclair, Antonia S. Stang, Matthieu Vincent, Terry P. Klassen

**Affiliations:** 1 Children’s Hospital Research Institute of Manitoba, 532-715 McDermot Avenue, Winnipeg, MB R3E 3P4 Canada; 2 Faculty of Nursing, University of Alberta, Edmonton, AB Canada; 3 Department of Pediatrics, University of Alberta, Edmonton, AB Canada; 4 Alberta Research Centre for Health Evidence, Edmonton, AB Canada; 5 Department of Pediatrics & Emergency Medicine, University of Ottawa, Ottawa, ON Canada; 6 Children’s Hospital of Eastern Ontario (CHEO) Research Institute, Ottawa, ON Canada; 7 Department of Pediatrics, University of Calgary, Calgary, AB Canada; 8 Department of Physiology and Pharmacology, University of Calgary, Calgary, AB Canada; 9 Department of Emergency Medicine, University of Calgary, Calgary, AB Canada; 10 Department of Emergency Medicine, Dalhousie University, Halifax, NS Canada; 11 Department of Paediatrics, Faculty of Medicine, McGill University, Montreal, QC Canada; 12 Faculty of Medicine, Sherbrooke University, Sherbrooke, QC Canada; 13 Department of Pediatrics and Child Health, University of Manitoba, Winnipeg, MB Canada

**Keywords:** Pediatrics, Emergency, Knowledge translation, Knowledge mobilization, Evidence-based

## Introduction

In Canada, up to 85% of children who need emergency care are treated in general emergency departments (ED) [1] that care for children and adults. Children have unique healthcare needs due to physiological, development and psychological differences. ED teams in these general ED settings have identified gaps in accessing pediatric-specific training and resources, including opportunities for hands-on practice during courses or training experiences [2–4]. Moreover, they have expressed concerns in maintaining pediatric expertise and competencies particularly if they do not often treat children or certain pediatric conditions [2, 4]. These concerns are well-founded; the gap in pediatric care between pediatric and general EDs has been associated with a disparity in health outcomes [5]. Efforts are needed to improve ED pediatric readiness, and ensuring children have access to timely, well-resourced, and effective emergency care [6].

Families also face challenges making decisions on how best to care for their acutely ill or injured child. Health information filled with medical jargon and no consideration of parental literacy skills and language competencies is less effective [7]. Information needs of families must be prioritized to support their decision-making, shape treatment expectations, and optimize healthcare utilization [8, 9].

Canada has been at the forefront of pediatric emergency medicine research since 1995 when *Pediatric Emergency Research Canada (PERC)* was created [10]. PERC has been instrumental in advancing pediatric emergency knowledge in numerous clinical areas [10]. PERC was also a founding member of the international *Pediatric Emergency Research Networks* (PERN), which has led observational studies and clinical trials for topics, including sepsis, H1N1, COVID-19, and pneumonia [11]. However, unless research findings are effectively implemented into clinical practice within *all* EDs that treat children, we cannot truly bridge the ‘research-to-practice’ gap and raise the overall standard of emergency care for children.

In 2011, *Translating Emergency Knowledge for Kids* (TREKK) was established to address this research-to-practice gap. Supported by the Government of Canada’s Networks of Centres of Excellence Knowledge Mobilization (NCE-KM) initiative, infrastructure was built that initially connected 12 PERC sites (based in pediatric institutions) [10] with 37 general EDs in nine provinces/territories. Together, clinicians and researchers at these sites set out to accelerate the speed at which the latest evidence in pediatric emergency care was shared with two key receptor communities: (1) healthcare professionals working within general EDs; and (2) parents/families seeking emergency care for their child. The goals were to: (1) determine the knowledge needs of our receptor communities; (2) assemble evidence and develop educational tools to help general EDs access, adapt and implement new knowledge; and (3) build a sustainable knowledge mobilization network in pediatric emergency care.

## Determining knowledge needs

TREKK began with a national needs assessment that surveyed over 1400 general ED clinicians and 1000 parents in 32 general ED sites (rural, remote and urban) across Canada to identify their pediatric information needs/preferences and more fully understand the context of care delivery at these sites [2]. In-person survey data collection was followed by focus groups with ED healthcare professionals to discuss the survey findings. A review of pediatric transport data, coroner reports and medical claims explored unperceived information needs. Some of the pediatric topics of greatest interest to healthcare professionals (e.g., multi-system trauma, meningitis, and severe head injuries) were infrequent but high stakes events. Needs assessment findings were fundamental in prioritizing topics and knowledge mobilization strategies.

## Developing educational tools and resources

**Fig. 1:**
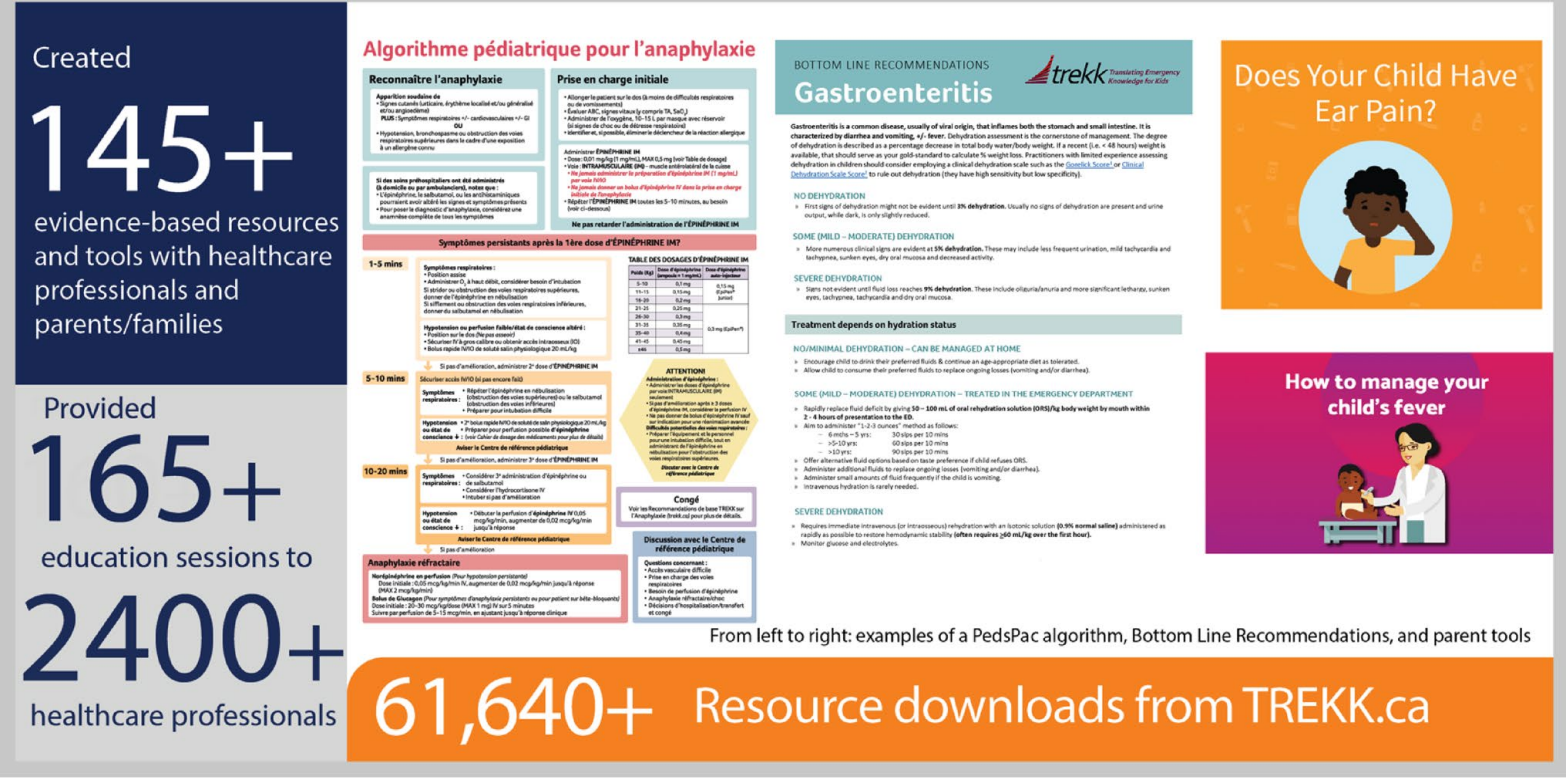
Examples of TREKK resources and reach Source: Adaptations to resources/tools or use of images require permission

TREKK established resource development infrastructure that brings together evidence synthesis experts, leading child health researchers, communication specialists and clinicians within a variety of specialties to identify, review, curate and create evidence-informed resources [12]. The TREKK website has links to vetted, existing resources. When a gap exists, TREKK develops new resources. To date, over 145 TREKK resources have been developed, including:

- Pediatric Packages (PedsPacs)—comprehensive bundles of practical tools (e.g., management algorithms, pocket cards, order sets, transport checklist, triage screening poster) on a range of topics (Fig. 1). When a lack of evidence exists for specific treatment recommendations, guidance is based on the consensus of pediatric experts across Canada.
- Bottom Line Recommendations (BLR)—1–2-page summaries that highlight key issues and offer a review of current, available evidence for managing children in emergency settings. The BLRs (Fig. 1) are not intended as step-by-step guides, rather they summarize existing evidence and provide practical information on a given pediatric emergency topic.

Resources are available in French and English, updated every two years at minimum, and openly accessible via the TREKK website (Trekk.ca) and app (Trekkca), allowing clinicians ready access at any time. The TREKK app was developed with over 30 healthcare professionals and purposefully designed to store resources offline for use in remote settings with no internet access. The development of these novel resources represents a national effort to reduce delays and improve efficiencies in evidence uptake. For example, when a large multicentre pediatric DKA trial was underway [13], TREKK was in regular contact with the investigators and started modifying its DKA resources. Once the results were published, TREKK released the updated resources. Changes in practice that would typically take years to implement were reduced to months, allowing emergency clinicians across Canada to implement new evidence immediately.

TREKK researchers also co-create resources *with* families (Fig. 1) through a process that merges evidence, parents’ stories, art, and digital media to share information on common childhood conditions. Parents are actively involved in the development process, which includes parent interviews, systematic reviews, prototype development with professional writers and media designers, review by healthcare professionals for clinical accuracy, review by parents for content and aesthetics, and usability testing [14]. The parent tools are available online and within ED waiting rooms and health centres in several provinces, reaching millions of families.

Flexibility to adapt TREKK resources to local contexts prevents developing resources ‘in silos’, reduces duplication of effort, and gives healthcare professionals more time to focus on clinical care and training. A collaboration with Indigenous Services Canada led to modification of TREKK’s sepsis and status epilepticus resources, to make them more applicable to nursing station use. In addition, TREKK resources have been adapted by the Emergency Medical Services for Children (EMSC) Innovation and Improvement Center (EIIC) for use in EDs in the United States (https://emscimprovement.center/about/). Collaboration between TREKK and EIIC has new resources being jointly created for use by the two organizations.

TREKK resources have had 61,640 downloads from trekk.ca as of August 2021. TREKK teams have facilitated 165 education sessions with general EDs across Canada, reaching over 2400 physicians, nurses, respiratory therapists, pharmacists, and paramedics. Whenever possible, these education sessions occur in-situ within general EDs and use simulation to identify environmental and systemic issues, such as equipment concerns, medication availability, and team dynamics. Virtual education sessions have taken place during the COVID-19 pandemic, and a longer term virtual education platform is being explored.

## Sustaining pediatric emergency knowledge mobilization

TREKK is a not-for-profit organization governed by a national Board of Directors, fueled by the in-kind support of over 40 steering, editorial and advisory committee members, and supported by staff at the Children’s Hospital Research Institute of Manitoba (CHRIM). The NCE funding program ended in 2020. Generous support from Children’s Hospital Foundations allows TREKK’s central operations to continue. A collaboration with the Manitoba Métis Federation is supporting the cultural adaption of parent tools. Grant funding is supporting specific projects, such as developing parent tools [14] and assessing how ready EDs are to treat children. However, infrastructure that accelerates access to evidence and linkages to expertise (e.g., researchers, knowledge brokers, and librarians) and people with lived experiences (e.g., parents/families) are foundational for enabling, sustaining, and evaluating consistent evidence-informed health care [15]; funding for such infrastructure is not available through traditional granting mechanisms.

To scale up TREKK’s impact and reach all general EDs and nursing stations in Canada will require appropriate investment in both human and financial resources. Within the US, this infrastructure has been supported by federal funds and legislation for over 30 years (https://emscimprovement.center/about/) and has shown that general EDs can improve how ready they are to manage a sick or injured child when high-quality resources and best practices are shared [5, 6]. Investment in a pan-Canadian strategy for pediatric emergency knowledge mobilization is needed, whereby health systems can work together to focus on common problems while maintaining independence to address issues relevant to local contexts. Without this investment, the potential for disparity in healthcare outcomes and quality within pediatric emergency care will continue to exist.

## Conclusion

TREKK’s collaborative approach has determined the information needs of both emergency healthcare professionals and families. TREKK’s infrastructure provides instant, online access to thousands of pediatric emergency resources, including Canadian developed, evidence-based clinical and educational resources. Education sessions, meetings and technology are building connections between over 165 rural, remote, and urban EDs across Canada. Scaling, spreading, and sustaining these achievements will require a move beyond collaborative relationships and the in-kind support of passionate clinicians, parents, and researchers. Sustained investment in TREKK, which integrates pediatric emergency knowledge within our health systems and supports the pediatric readiness of general EDs, is needed. Only then can we truly move towards equitable emergency care for all children in Canada.
